# Identifying Conformational Isomers of Organic Molecules in Solution via Unsupervised Clustering

**DOI:** 10.1021/acs.jcim.0c01387

**Published:** 2021-04-29

**Authors:** Veselina Marinova, Laurence Dodd, Song-Jun Lee, Geoffrey P. F. Wood, Ivan Marziano, Matteo Salvalaglio

**Affiliations:** † Thomas Young Centre and Department of Chemical Engineering, University College London, London WC1E 7JE, U.K.; ‡ Department of Materials Science and Engineering, The University of Sheffield, Sheffield S1 3JD, U.K.; § Pfizer Worldwide Research and Development, Groton Laboratories, Groton, Connecticut 06340, United States; ∥ Pfizer Worldwide Research and Development, Sandwich CT13 9NJ, Kent, U.K.

## Abstract

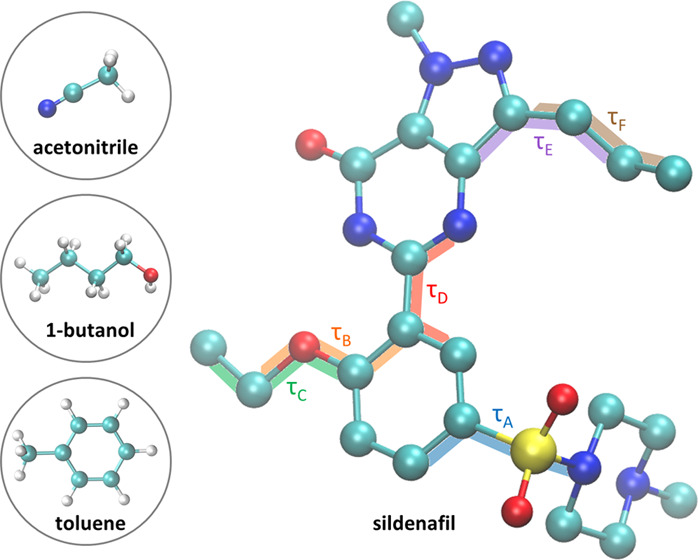

We present a systematic approach for the identification of statistically relevant conformational macrostates of organic molecules from molecular dynamics trajectories. The approach applies to molecules characterized by an arbitrary number of torsional degrees of freedom and enables the transferability of the macrostates definition across different environments. We formulate a dissimilarity measure between molecular configurations that incorporates information on the characteristic energetic cost associated with transitions along all relevant torsional degrees of freedom. Such metric is employed to perform unsupervised clustering of molecular configurations based on the Fast Search and Find of Density Peaks algorithm. We apply this method to investigate the equilibrium conformational ensemble of Sildenafil, a conformationally complex pharmaceutical compound, in different environments including the crystal bulk, the gas phase, and three different solvents (acetonitrile, 1-butanol, and toluene). We demonstrate that while Sildenafil can adopt more than 100 metastable conformational configurations, only 12 are significantly populated across all of the environments investigated. Despite the complexity of the conformational space, we find that the most abundant conformers in solution are the closest to the conformers found in the most common Sildenafil crystal phase.

## Introduction

Conformational isomerism in organic molecules is an important characteristic that bears significance in a variety of problems. For example, binding properties of proteins in protein–ligand complexes are controlled by their conformational configuration by affecting association/dissociation rates and by entropic contributions to the process.^1,2^ Understanding the details and mechanisms of conformational changes that proteins undergo is an important part of modern drug discovery methodologies.^3^ For small organic molecules, the ability to adopt different conformational configurations can open the possibility for the formation of multiple crystal forms known as conformational polymorphs^4,5^—crystal structures of components with the same chemical formula but different molecular shapes. This phenomenon is particularly important in the pharmaceutical industry where the uncontrolled occurrence of an undesired polymorphic form can affect the stability, shelf-life, or efficacy of the drug. In the field of crystallization, conformational rearrangements are not only relevant to polymorphism. In our previous work^6^ on the study of ibuprofen conformational isomerism at the crystal/solution interface, we demonstrate how, even for relatively small systems, conformational rearrangements, crystal growth, and dissolution are inherently coupled. Additionally, state-to-state transitions of a molecule along its path of incorporation into the crystal from solution may be limited by conformational rearrangements.

Computational studies of conformational rearrangement in small organic molecules often use internal torsional angles to describe the adopted molecular configuration.^6−9^ Torsional angles are a convenient way of describing rearrangements as they provide a fine-grained comprehensive picture of the internal molecular configuration space. To describe the conformation of larger molecules such as peptides or aliphatic chains, however, resorting to descriptors such as end-to-end distance or root-mean-square deviation (RMSD)^1,10^ is a common choice, made necessary by the fact that the torsional angle space for these systems is high-dimensional and impractical to read and interpret. A critical drawback of this approach is that by reducing the dimensionality of the descriptors’ space used to represent configurations, degeneracy is introduced, and consequently information is lost. More generally, reliable conformational descriptors are particularly important when implementing enhanced sampling techniques. Enhanced sampling techniques are heavily reliant on the use of appropriate system descriptors. Particularly for studying self-assembly processes, conformationally flexible systems currently present a major challenge,^11^ thereby driving the search for a systematic approach to their classification.

Dividing the conformational space of large organic molecules is often done via partitional clustering methods, meaning that the data is assigned into groups without any hierarchical structure, based on a chosen criterion.^12^ One of the best-known partitioning algorithms is *k*-means.^13^ The idea behind this algorithm is to define a *k*-centroid for each cluster and measure the distance between a data point and each of the cluster centers. Many computational works have achieved partitioning of molecular configurational space through *k*-means clustering-based methodologies.^14−18^ Despite its ease of use, it has a few important limitations. Cluster centers can be difficult to define *a priori*, as well as, *k*-means can be very sensitive to outliers and noise.^19,20^ A partition-based algorithm that has tackled the drawbacks of *k*-means clustering is affinity propagation (AP),^21^ where all data points are regarded as potential cluster centers. The negative distance between data points is their affinity, so the bigger the sum of the affinity of one data point to other data points, the higher the probability of this data point being a cluster center is. AP has been implemented in the study of protein conformations;^22^ however, it has also been regarded as a complex and costly approach.^19,23^

As an alternative to distance-based algorithms, density-based algorithms have been developed.^20,24−28^ They work on the principle of assigning densities to local points and are able to separate clusters based on high- and low-density regions. Such algorithms not only do not require defining the number of cluster centers *a priori* but also allow the identification of nonspherical clusters. Particularly suited to the analysis of molecular dynamics (MD) simulations is the Fast Search and Find of Density Peaks (FSFDP) clustering algorithm, developed by Rodriguez and Laio.^26^ By computing the distance between all pairs of data points, the algorithm identifies the points with the highest density in their neighborhood as the cluster centroids.

Such tools, which allow the systematic classification of molecular configurations regardless of the dimensionality of the space of descriptors necessary to completely capture every conformational change, have the potential to improve existing methodologies for studying the effects of conformational rearrangements during crystal nucleation and growth.^6^

Here, we propose a methodology that enables the study of conformational isomerism in a general way for systems with a large number of torsional degrees of freedom. Our approach, based on the application of the Fast Search and Find of Density Peaks clustering algorithm,^26^ allows defining a set of conformational states that is common to multiple environments (i.e., solvents) and enables a systematic assessment of their impact on the conformational landscape. We demonstrate this approach by studying the conformational rearrangement of sildenafil, a commercially available active pharmaceutical ingredient (API).^7^

Sildenafil is the main component of Viagra,^7^ which is known to have two polymorphic forms.^29,30^ Sildenafil is a relatively large molecule consisting of 63 atoms and a number of ring structures. The two forms of sildenafil (denoted form I and form II) are morphotropically related to one another as a noncrystallographic rearrangement can transform one to the other,^30^ with form I being the thermodynamically stable form. Both forms have two molecules in the asymmetric unit adopting different conformations.^29,30^

With the use of a data clustering approach, we demonstrate how characteristic conformational configurations can be identified *a priori* for a molecule in the gas phase to then extract quantitative information on conformational states from enhanced sampling molecular dynamics simulations performed in solution under experimentally relevant conditions. Our methodology also enables the breakdown of the free energy of a conformational state into enthalpic and entropic contributions, providing a valuable insight into the effect of the solvent on conformational isomerism. With this work, we aim to propose a method for conformational analysis which provides a route toward achieving rapid and automated conformational classification, enabling the comprehensive study of conformational isomerism in solution for systems for which it is currently impractical.

## Methods

In this work, molecular dynamics (MD) simulations are used to study the conformational isomerism of sildenafil in the crystal bulk, in the gas phase, and in three solvents—acetonitrile, 1-butanol, and toluene. MD is combined with well-tempered metadynamics (WTmetaD) to enable the study of the conformational rearrangements of sildenafil in solution. The Fast Search and Find of Density Peaks (FSFDP) clustering method, developed by Rodriguez and Laio,^26^ is used to identify sildenafil conformers in the gas phase and generate a characteristic fingerprint in torsional angle space for each of them. The metric used to define the similarity between configurations includes information on the free energy cost associated with transitions in every degree of freedom explicitly considered. The fingerprints are used to postprocess the biased trajectories in solution and assign a conformational macrostate for each trajectory step. Through the implementation of a reweighting procedure, the equilibrium probability of conformers, as well as enthalpic and entropic contributions to the free energy of each conformer for each of the solvents considered, is obtained.

**Figure 1:**
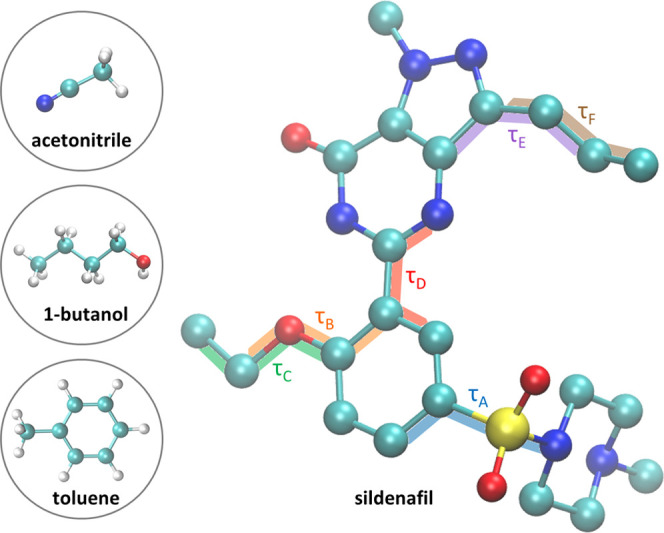
Sildenafil structure, where hydrogen atoms have been excluded for simplicity. The six internal torsional angles, labeled τ_A_, τ_B_, τ_C_, τ_D_, τ_E_, and τ_F_, are marked on the structure. All images of molecular structures shown have been generated with visual molecular dynamics (VMD).^31^

In this study, the molecular rearrangement of the drug is described in torsional angle space by considering all internal dihedral angles, as shown in Figure 1. By including all torsional degrees of freedom of the molecule, this study adopts a systematic and transferable strategy for tackling conformational rearrangement.

### Molecular Dynamics Setup

Molecular dynamics simulations of form I and form II crystal polymorphs of sildenafil, a sildenafil molecule in the gas phase, and a sildenafil molecule in three different solvents were performed using the Generalized Amber Force Field (GAFF).^32^ For all systems considered in this work, GAFF is able to reproduce properties consistent with experimental data (see the Supporting Information). MD simulations were performed with Gromacs 5.1.4^33^ with an explicit representation of the solvent. Force field parameters for solvent molecules were obtained from the Virtual Chemistry solvent database.^34,35^ A standard cut-off distance of 1.0 nm for the nonbonded interactions was chosen, along with including long-range intermolecular interactions using the particle-mesh Ewald (PME) approach.^36^ A time step of 2 fs was used. Temperature and pressure control have been implemented through the use of the Bussi–Donadio–Parrinello thermostat,^37^ Berendsen barostat,^38^ and Parrinello–Rahman barostat.^39^ More detail on the applied pressure control and the recovered system density in each case can be found in the Supporting Information.

#### Simulations of the Crystal Bulk

Supercells of size 3.5 × 3.5 × 5.0 and 7.0 × 3.5 × 2.3 nm^3^, representing crystal forms I and II, respectively, were set up containing 96 molecules each. Crystal structure.cif files^29^ at ambient temperature and pressure were obtained from the Cambridge Structural Database (CSD) under deposition codes QEGTUT and QEGTUT02. In both cases, unbiased MD simulations were performed for 10 ns in the isothermal–isobaric ensemble, implemented by applying an anisotropic pressure control.

#### Simulation of Sildenafil in the Gas Phase

A 450 ns unbiased simulation of a sildenafil molecule in a box of 2.0 × 2.0 × 2.0 nm^3^ in a vacuum was carried out in the canonical ensemble. A free energy profile of each torsional angle as denoted in Figure 1 was obtained. All one-dimensional free energy profiles are reported in the Supporting Information.

#### Simulations of Sildenafil in Solution

Simulations in solution were set up by solvating a single sildenafil molecule with each of the three solvents used in this study—acetonitrile, 1-butanol, and toluene—using the insert-molecules utility in Gromacs in a box of an approximate size of 4 × 4 × 4 nm^3^. MD simulations were performed in combination with well-tempered metadynamics. All simulations were performed in the isothermal–isobaric ensemble (NPT) at a pressure of 1 bar and temperature of 300 K.

### Well-Tempered Metadynamics Setup

Metadynamics was implemented to enhance fluctuations in the internal rearrangement of sildenafil in solution for computational efficiency. The bias was applied as a function of the τ_A_ torsional angle as shown in Figure 1 in blue. The choice of the collective variables (CV) was made based on 10 ns exploratory MD simulations, which revealed that overcoming the barrier associated with the rotation of τ_A_ in an efficient way requires enhanced sampling in all three solvents. The biasing protocol was applied in the form of Gaussian functions with a width of 0.3 rad and height of 2.5 *k*_B_T at a rate of every 500 simulation steps with a bias factor of 15 K. The use of WTmetaD was implemented through plumed 2.4.^40^ All of the data and input files, required to reproduce the results reported in this paper, are available on plumed-NEST,^41^ the public repository of the PLUMED consortium as plumID:20.032.

### Clustering Procedure

Studying the conformational isomerism of systems with several internal degrees of freedom in a systematic and transferable way requires to group molecular configurations, identifying relevant conformational states. Achieving this provides the opportunity of reducing the dimensionality of the problem and enables the analysis of biased molecular dynamics trajectories to obtain useful kinetic and thermodynamic information.

To obtain this partitioning of configurational space in an unsupervised, data-driven manner we apply the Fast Search and Find of Density Peaks (FSFDP) algorithm, developed by Rodriguez and Laio.^26^ The algorithm is used to group molecular configurations into clusters of structures based on their similarity, defined by a distance matrix. The distance matrix contains the distance between any two molecular configurations, *i* and *j*, as a function of the chosen system descriptors, e.g., internal torsional angles, with no limit on dimensionality, as shown in eq 1
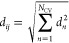
1Given a distance matrix, the algorithm operates by calculating the density of each point *i*, evaluated by considering the number of neighbors within a distance cutoff *d*_c_ according to
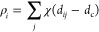
2where χ(*x*) = 1 if *x* < 0 and χ(*x*) = 0 otherwise
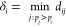
3Cluster centers are characterized by having the highest density within a cluster of points and a large distance from points with a higher density as shown in eq 3. Each cluster has an associated core set of data points and a halo, evaluated based on the given cutoff. The core set are the points that belong exclusively to a cluster and can be found within the selected distance cutoff from the cluster center, while the halo is considered as the noise around the cluster core while still affiliated with the given cluster. This grouping algorithm is particularly powerful as the clustering procedure is such that the number of clusters arises intuitively, which makes it particularly suited to identifying conformational states described by a highly multidimensional set of CVs.

Here, we consider each frame of an MD trajectory of a sildenafil molecule in the gas phase as a single point in the six-dimensional torsional angle space and so the distance *d*_*ij*_ between every two frames, *i* and *j*, is calculated according to eq4
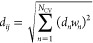
4where *N*_CV_ refers to the total number of system descriptors, which in this case is six torsional angles, while *w* stands for a weight applied to each dimension as described below.

In the above equation, *d*_*n*_ refers to the absolute difference in the values of any of the given torsional angles for points *i* and *j*. For example, *d*_1_ is calculated as
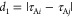
5Should the value of *d*_*n*_ be greater than π, periodicity is accounted for by subtracting the value from 2π as follows
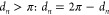
6Clustering structures in a meaningful way requires a distance definition able to distinguish between conformational transitions and conformational adjustments. These terms refer to the nature of the conformational rearrangement within the molecule. A conformational transition describes the conversion of one stable conformational state into another, usually associated with overcoming a free energy barrier higher than *k*_B_T. An adjustment is, on the other hand, a term used to describe a minor rearrangement, which is not associated with a new conformer, but rather with a relaxation of the structure from one configuration to another, both of which occupy the same free energy minimum in collective variable space.^5^ To resolve these two cases, when calculating the distance *d* a slight modification to the algorithm is introduced by including the weight factor *w* (see eq4), associated with the free energy barrier of rotation of each torsional angle. In such a way, rotations that lead to new conformational configurations through rare, activated transitions provide a larger contribution to the distance compared to rotations associated with an adjustment or a fast conversion.

**Figure 2:**
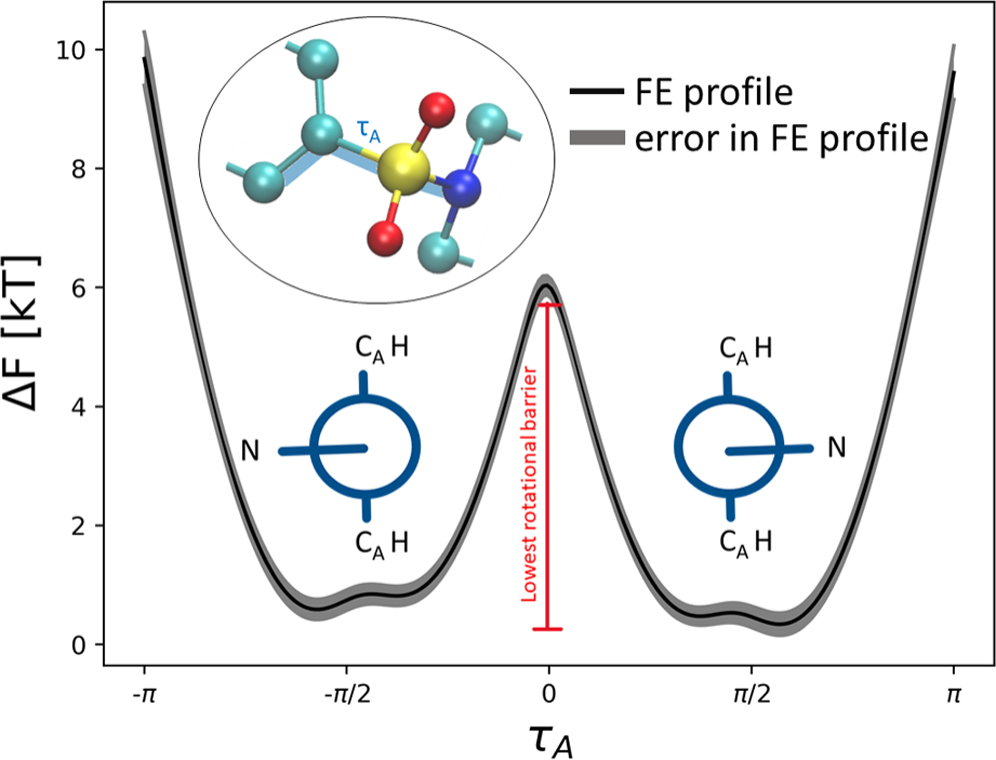
Free energy profile of torsional angle τ_A_ obtained from a vacuum simulation, along with Newman projections of the angle in the free energy minima. The lowest free energy barrier of rotation was extracted for the clustering weights.

The weight factor, *w*, is defined as the lowest rotational barrier for each torsional angle according to the calculated one-dimensional free energy profiles (see the Supporting Information) along each coordinate of the CV space. The barriers are then normalized with respect to the lowest one, as shown in Table 1. An example for the case of τ_A_ is shown in Figure 2.

**Table 1:** Free Energy Barrier of Rotation to Each Torsional Angle Obtained from Simulations in Vacuum, along with the Corresponding Rescaling Used as a Weight in Calculating the Distance Matrix

angle	barrier height	weight
	(*k*_B_T)	
τ_A_	6	5
τ_B_	1.5	1.3
τ_C_	2	1.7
τ_D_	1.2	1
τ_E_	2.5	2.1
τ_F_	4	3.3

We note that in the case of larger, more complex solutes with a higher number of conformational degrees of freedom one may need to analyze large data sets, and in such cases, the calculation of the distance matrix may become an efficiency bottleneck for the process. Recent work^27^ addresses this problem pushing the limit of applicability of FSFDP to the million-configuration range.

### Conformational Classification

Clustering of the molecular configurations was performed using the FSFDP algorithm, with a distance cutoff *d*_c_ tuned to obtain an average number of neighbors for every data point equal to 2% of the entire data set. Based on the distance matrix calculation and the chosen cutoff, the sildenafil configurations sampled in the gas phase are grouped into clusters, each consisting of a cluster center structure, a core set of configurations, and a halo. All configurations assigned to a cluster were used to generate a structural fingerprint for the identified conformer. The term fingerprint refers to the probability distribution of each torsional angle as illustrated in Figure 1. All fingerprints are provided in the Supporting Information.

Conformational classification of each frame of the trajectories of sildenafil in solution was carried out to represent a conformational change of the molecule in a one-dimensional space and hence enable further analysis of the enthalpic and entropic contributions to conformational isomerism in solution. To achieve that, an algorithm that compares the instantaneous value of the torsional angles, defined to describe the molecular configuration, to each of the given fingerprints for every trajectory frame was set up. For every instantaneous torsional angle value, where the corresponding probability density in the fingerprint is nonzero, a value of 1 is assigned. The total number of variables used for the classification is 6, and therefore a score of 6 means that the molecular configuration in the given frame matches a fingerprint and therefore is assigned the corresponding conformer number. A score lower than 6 indicates at least one mismatch between the given configuration and the fingerprint and is therefore assigned a value of 0, signifying that it remains unclassified.

### Conformational Equilibrium Probability Distribution

A characteristic fingerprint in torsional angle space for each dominating sildenafil conformer in the gas phase was generated. Choosing the gas phase as a reference is inspired by the work of Cruz-Cabeza and Bernstein,^5^ who use the same conditions to define reference conformational states. To calculate the conformational population of sildenafil in solution, each frame of the biased trajectory was assigned a characteristic conformer following the procedure discussed in the previous section. A discrete probability distribution in one-dimensional space can then be straightforwardly calculated, with the caveat that the bias potential deposited throughout the duration of the simulation needs to be accounted for in a procedure referred to as reweighting.

In this work, the total metadynamics bias potential applied as a function of τ_A_ and recovered at the end of the simulation of a sildenafil molecule in solution, *V*^total^(τ_A_), is used in the reweighing scheme.^42^ The trajectory is postprocessed so that each time frame, with a corresponding value for each torsional angle, as well a conformational cluster number, will also have a value associated with the total bias deposited in that particular point in the CV space of τ_A_. For simplicity, let us refer to this value as *V*_*i*_^total^(τ_A_), where *i* stands for the trajectory frame number. The weight *W*_*i*_ applied to each frame when reconstructing the unbiased probability distribution of conformational isomers in solution is a Boltzmann weight associated with a rescaled value of *V*_*i*_^total^(τ_A_) according to
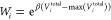
7In such a way, the weight associated with points in CV space, where the maximum total external bias was deposited, will have a value of 1, as it corresponds to the lowest point in the free energy profile in τ_A_ and all other frames will have a correspondingly lower weight. This reweighting scheme was implemented to reconstruct the population of sildenafil conformers in different solvents, as well as obtaining a two-dimensional probability density function of the conformer number and its associated potential energy, used in the free energy decomposition discussed in the next chapter.

### Enthalpy and Entropy Contributions to Free Energy Differences between Clusters

Free energy differences between conformational macrostates *i* and *j* of sildenafil in solution were computed from their equilibrium probability *_Pi_* and *P*_*j*_ as  and decomposed into their enthalpic and entropic contributions following the procedure outlined by Gimondi et al.^43,44^ For instance, the difference in free energy between clusters *i* and *j*, Δ*G*_*i*,*j*_, can be expressed as Δ*H*_*i*,*j*_ – *T*Δ*S*_*i*,*j*_, where Δ*H*_*i*,*j*_ and Δ*S*_*i*,*j*_ are the enthalpy and entropy differences between states *i* and *j*. Hence, the entropic contribution can be obtained by difference as *T*Δ*S*_*i*,*j*_ = Δ*G*_*i*,*j*_ – Δ*H*_*i*,*j*_, once the term Δ*H*_*i*,*j*_ is known. Since conformational transitions of sildenafil are not associated with a change in the ensemble average of the system’s volume, Δ*H*_*i*,*j*_ = Δ*U*_*i*,*j*_ + *P*Δ*V*_*i*,*j*_ ≃ Δ*U*_*i*,*j*_, where Δ*U*_*i*,*j*_ is the difference in internal energy between conformational macrostates *i* and *j*. Moreover, since states *i* and *j* are conformational isomers sampled at the same temperature, the internal energy difference reduces to the difference in the ensemble average of the potential energy between states *i* and *j* as
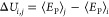
8where ⟨*E*_P_⟩_*i*_, the ensemble average of the potential energy of configurations classified in cluster *i*, is computed as
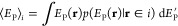
9where *p*(*E*_P_(**r**)|**r** ∈ *i*) is the potential energy probability density, conditional to the system being classified in cluster *i*. The probability density *p*(*E*_P_(**r**)|**r** ∈ *i*) is computed as discussed by Gimondi et al.,^43^ using the reweighting strategy described in the previous paragraph. In models in which explicit solvents are employed, ⟨*E*_P_⟩_*i*_ is typically dominated by the contribution of the solvent molecules, and it is associated with large fluctuations that affect the convergence and accuracy of the Δ*U*_*i*,*j*_ estimate. To improve the statistical accuracy in Δ*U*_*i*,*j*_, we follow the procedure outlined by Kollias et al.^44^ and decompose the ensemble average of the potential energy in three components, namely ⟨*E*_P_⟩_*i*_^solute^, ⟨*E*_P_⟩_*i*_^solvent^, and ⟨*E*_P_⟩_*i*_^solute–solvent^. The ⟨*E*_P_⟩_*i*_^solute^ and ⟨*E*_P_⟩_*i*_^solvent^ contributions account, respectively, for potential energy terms associated with interactions between atoms that belong exclusively to the solute and to the solvent species. The solute–solvent term ⟨*E*_P_⟩_*i*_^solute–solvent^ accounts instead for nonbonded interactions between solute and solvent atoms. Since conformational changes in the solute do not affect the solvent–solvent contribution to the potential energy, the term ⟨*E*_P_⟩_*i*_^solvent^ = ⟨*E*_P_⟩_*j*_^solvent^ = const, and thus Δ*U*_*i*,*j*_ reduces to
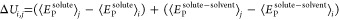
10which is not affected by the large fluctuations of ⟨*E*_P_⟩_*i*_^solvent^ that would mask the contribution of conformational transitions to Δ*U*_*i*,*j*_ and hamper the convergence of the enthalpy and entropy contribution to free energy differences. Despite implementing this strategy, the convergence of the enthalpic and entropic contributions require a substantial sampling of the configuration space of the explicitly solvated system. Here, we achieve sufficient sampling by running WTmetaD simulations for 0.5–0.6 μs. In the case of larger, more complex solutes with a higher number of conformational degrees of freedom, we anticipate the need for replica exchange methods to exhaustively sample the configuration space and successfully apply this decomposition approach.

## Results

The following sections summarize the analysis carried out on sildenafil conformers in the gas phase, as well as on the conformational rearrangements of sildenafil occurring in the crystal bulk and in solution. The results are organized as follows. First, the conformational freedom of sildenafil in the crystal bulk is reviewed by considering the torsional angle distribution obtained from MD simulations of forms I and II. Next, the results obtained from the clustering algorithm are reported, along with drawing a comparison between structures in the gas phase and those in the solid. The last section reports on the equilibrium distribution of sildenafil conformers in different solvents, obtained with the aid of WTmetaD, along with a breakdown of their corresponding free energy into enthalpy and entropy contributions.

### Conformational Rearrangements in the Crystal Bulk

The conformational rearrangement of sildenafil was first investigated for the case of a molecule in the crystal bulk in each of the two polymorphic forms. As mentioned, each polymorph contains two conformational isomers of the molecule. A probability distribution of each torsional angle representative of the conformational state adopted by sildenafil in the solid was obtained, which enables to gain insight into the degree of conformational freedom available in each crystal form.

**Figure 3:**
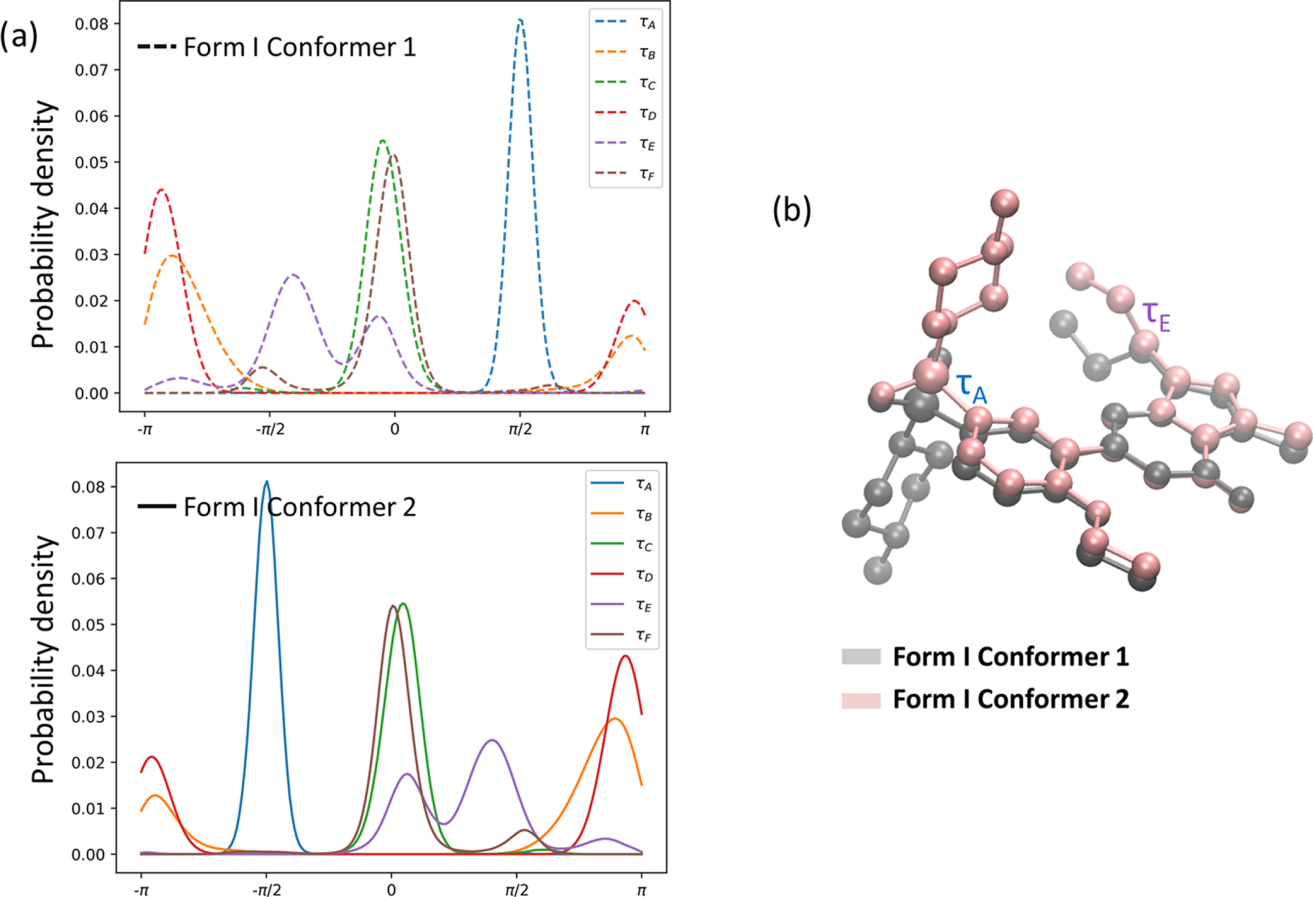
(a) Probability density plots of each torsional angle for crystal conformers 1 and 2 obtained from a 10 ns MD simulation of form I sildenafil. (b) Image of crystal form I conformers 1 and 2, obtained from the.cif, generated with VMD, where hydrogen atoms are removed for simplicity.

The results reveal that while several of the dihedral angles of sildenafil are completely restrained by the crystal packing, a surprising amount of flexibility is accessible to the rest. A probability distribution of each torsional angle for crystal conformers 1 and 2 in form I is shown in Figure 3a. In the figure, a narrow and mono-modal distribution corresponds to each of the torsional angles τ_A_, τ_C_, and τ_F_, shown, respectively, in blue, green, and brown. A mild degree of conformational adjustment is associated with torsional angles τ_B_ (in orange) and τ_D_ (in red), which are both distributed around ±π. These torsional angles are associated with adjacent substituents on the phenyl ring, suggesting that the rearrangement is possibly related to relieving steric hindrance. The highest degree of rotational freedom is observed in the case of torsional angle τ_E_, shown in purple, representing the rotation of the propanyl substituent of the pyrazole ring (see Figure 1), displaying a multimodal distribution. The flexibility of τ_E_ reveals a moderate degree of conformational rearrangement available to the conformer, despite the restrictive environment traditionally associated with a crystal. This observation has been addressed by Barbas et al.^29^ who have recorded the presence of a dynamic disorder in the propyl groups at room temperature in form I, which disappears at temperatures lower than 100 K. The authors justify this observation with the fact that these functional groups do not establish strong intermolecular interactions with the surrounding atoms within the crystal, resulting in a moderate degree of flexibility in the chain.

Analyzing the torsional angle distributions of each of the two conformers found within the crystal structure of form I reveals that they are conformational isomers, where a rotation along τ_A_ and τ_E_ can convert conformer 1 into conformer 2, as shown in Figure 3b. According to the results obtained from the MD simulation of form I, the internal rearrangement of crystal conformer 2 displays an identical behavior as to that of conformer 1.

Similarly, the conformational freedom of sildenafil in form II was analyzed through an unbiased MD simulation. A comparison of the probability distribution of each torsional angle of sildenafil between form I and form II reveals a similar conformational configuration in the two structures, with the only difference being a marginally lower flexibility of τ_E_ in form II compared to that observed in form I. A detailed comparison between the conformers in each form is provided in the Supporting Information. An experimental comparison between the two crystal forms is provided by Barbas et al.,^30^ who make a similar observation to the one reported here and stress that any differences between conformers in the two crystals can be classified as conformational readjustments of the same gas-phase conformer, validating the conclusions made on the basis of our MD simulations. As mentioned, the probability distribution of torsional angle τ_E_, associated with the rearrangement of the pronanyl group, shows that the degree of rearrangement is counterintuitively lower in form II compared to form I, despite the presence of larger structural voids in the former. The experimental publication does not report measurements of the degree of disorder in form II; however, the authors speculate that the conformational rearrangement of the propanyl groups will be dominated by a drive to keep the cavities in the structure empty, which could explain the conformational behavior observed in the MD simulation of form II.

### Structure Clustering in the Gas Phase

This section reports on the results obtained from implementing a data clustering algorithm to group structures from an MD trajectory of sildenafil in the gas phase and identify characteristic conformational configurations. A trajectory of a molecule in a vacuum was chosen for this purpose, as in the absence of solvent effects, the internal rearrangement of the molecule is unhindered and thorough sampling of all possible molecular configurations can be achieved efficiently. This allows considering the full conformational space of sildenafil in the clustering procedure. Such an approach has the potential to provide a more meaningful and robust method of identifying representative conformational isomers over methods that rely on generating conformers through random search and local minima strategies. By considering the free energy profile of each torsional angle in the gas phase, as discussed in the Supporting Information, all possible combinations of structural local minima of the molecule in torsional angle space are estimated to be 144. However, in reality, each local minimum comprises an ensemble of configurations, meaning that 144 structures are a rather conservative estimate, and in practice, there is a swarm of possible stable molecular configurations. For that reason, failing to explore the collective variable space thoroughly encounters the risk of missing out important structures due to the sheer number of available configurations, even for systems of moderate flexibility.

**Figure 4:**
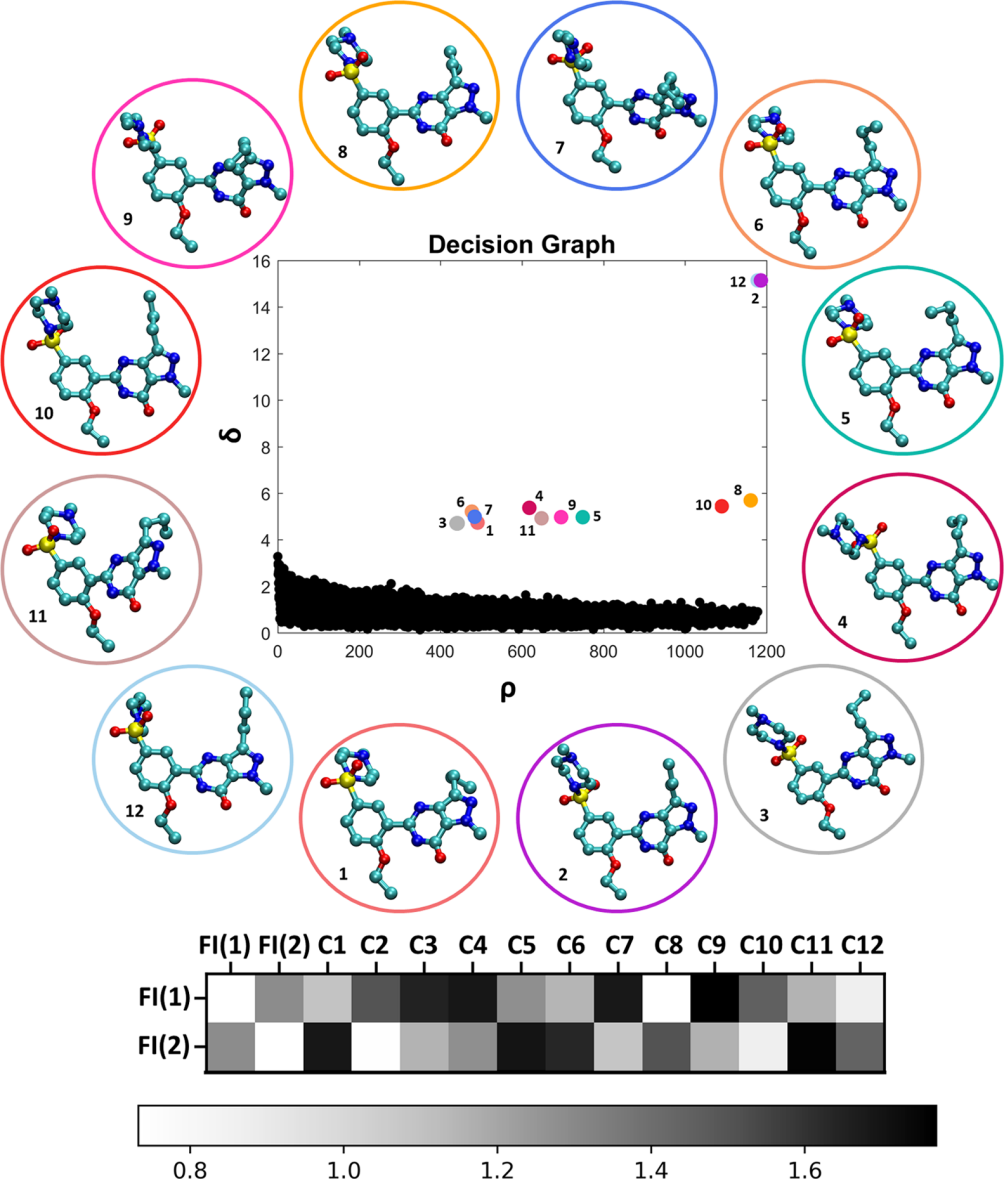
Decision graph of the structure clustering performed on the trajectory in the gas phase. The circled structures represent the cluster centers. The figure below represents the distance matrix between distributions of conformers 1 and 2 in the crystal and identified from the algorithm cluster centers.

A typical output of the clustering algorithm is a decision graph, displaying all data points as a function of their density ρ (number of neighbors) and distance from the nearest point of higher density δ (see eqs 2 and 3). When applied, the clustering algorithm determined 12 cluster centers, as shown in the decision graph in Figure 4. Each cluster center corresponds to the most representative conformer structure for those belonging to a cluster (core and halo). A visual representation of the molecular shape corresponding to each cluster center is displayed around the decision graph. The conformers identified at this stage are labeled C1–C12, each of which has an associated characteristic fingerprint in torsional angle space as discussed in the Methods section. All fingerprints can be found in the Supporting Information.

Before proceeding further into the analysis of the conformational population of sildenafil in solution, it is useful to compare the structures of the cluster centers identified in a vacuum and those of the crystal conformers. To this aim, a distance matrix comparing crystal conformers 1 and 2 found in form I and each of the 12 cluster center structures is generated as shown in Figure 4. In the plot, the color scale corresponds to the distance in torsional angle space, where white signifies the lowest distance, i.e., the most similar structures. The distance matrix was obtained by taking the most probable value for each angle and calculating the distance between points in the 6D space of all dihedrals.

**Figure 5:**
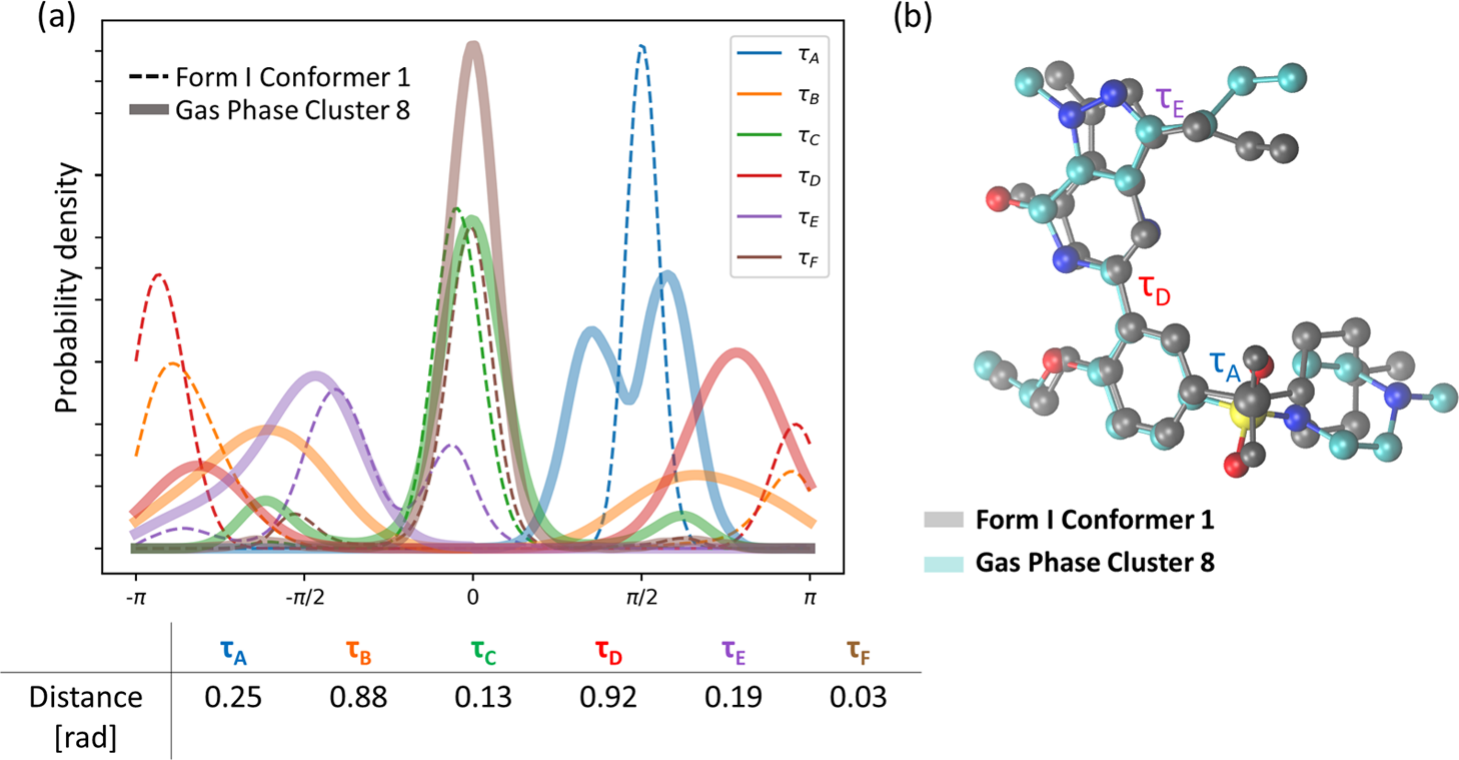
(a) Probability density of each torsional angle for crystal conformer 1 (dashed lines) and cluster center 8 (thick line). The numerical distance between distribution peaks is provided in the table below. (b) Comparison of crystal conformer 1 structure and the representative structure for C8 cluster group.

The distance matrix reveals that conformer C8 is the most similar structure to crystal conformer 1, while C2 is the most similar to conformer 2. The fingerprints corresponding to C8 and conformer 1 structures in torsional angle space are visually compared in Figure 5a. The two plots show that the conformational rearrangement of C8 into crystal conformer 1 would involve readjustment in torsional angles τ_A_, τ_B_, and τ_D_, which display a comparatively broader distribution in a vacuum. The overlap in the distributions demonstrates that the exact crystal conformer is found in the gas phase, and it accounts for only 3% of all configurations. Figure 5b shows a comparison between the cluster center structure of C8 and crystal conformer 1 as taken from the CCDC database, prior to performing MD. The overlap demonstrates the readjustment in τ_A_ and τ_D_ necessary to convert C8 into conformer 1. Torsional angle τ_E_ differs significantly according to the figure; however, as discussed, it has moderate flexibility in the solid and it relaxes to a configuration closer to that of C8 during MD, as shown in the probability distribution in Figure 5a in purple.

The second most similar to the crystal group of structures are conformers C10 and C12, which relate to, respectively, conformers C2 and C8 via a rotation of torsional angle τ_E_.

### Conformational Isomers of Sildenafil in Solution

#### Equilibrium Probability

MD simulations in combination with WTmetaD were used to investigate the conformational rearrangement of sildenafil in three different solvents—acetonitrile, 1-butanol, and toluene. The biased MD trajectory of each solvent case was analyzed using the characteristic fingerprints associated with structure clusters C1–C12, following the procedure outlined in the Methods section. The probability of each conformer cluster was generated by accounting for the deposited bias potential through the reweighting procedure discussed earlier.

**Figure 6:**
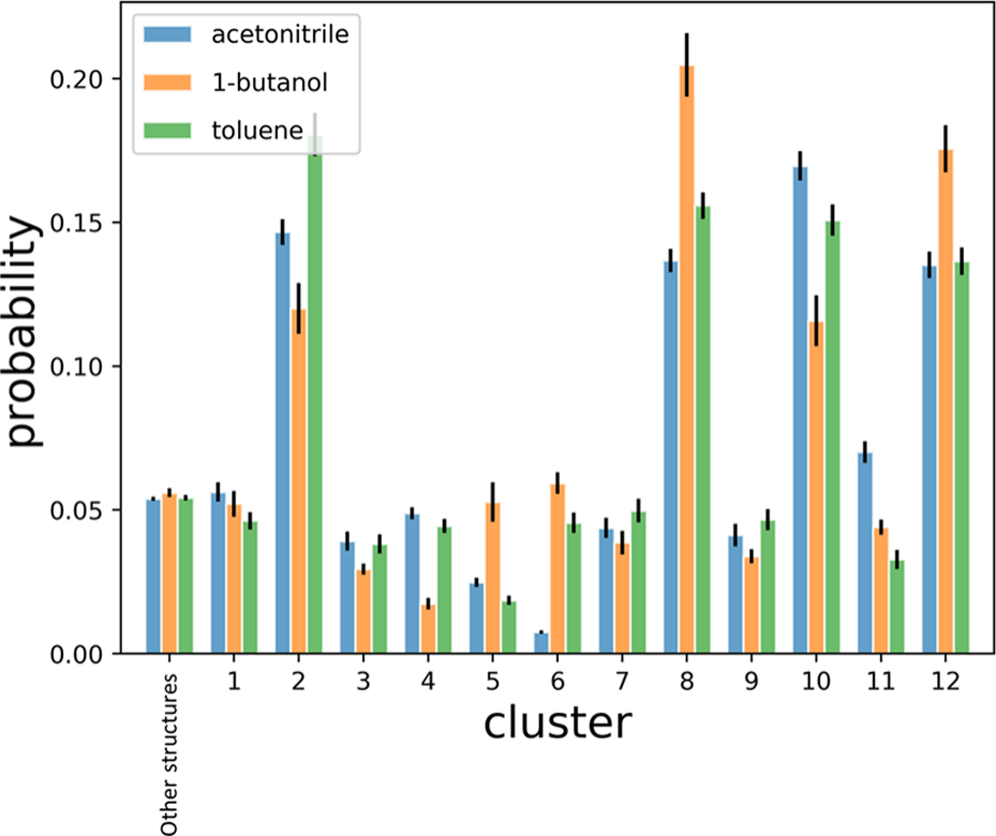
Probability of each conformer configuration C1–C12 along with crystal conformer 1 and crystal conformer 2 for all three solvents.

The obtained probability for all solvent cases is shown in Figure 6. The results show that 95% of configurations in solution are accounted for, indicating that the proposed procedure of identifying conformational structures via unsupervised clustering is a fast and reliable way of determining conformational configurations in solution for systems with a significant degree of conformational complexity. Examining the distributions, small but significant variations in the probability in different solvents are observed. These findings correlate with what was observed in our previous work for the case of ibuprofen.^6^ Structure types C2 and C8 are each found to account for 15–20% of the structures in solution. As discussed, these two groups represent the conformers with a structure closest to the crystal-like configuration of sildenafil. Furthermore, C10 and C12, which relate to C2 and C8 via a rotation along the fast-converting torsional angle τ_E_, each account for a further 15% of conformational isomers in solution. Therefore, given the high flexibility of τ_E_ even within the crystal structures, overall 60–70% of the structures found in solution resemble configurations close to those observed in the crystal, inferring that at the crystal–solution interface 30% of structures will have to encounter a more significant conformational rearrangement to adopt a crystal-like configuration and promote crystal nucleation or growth.

#### Enthalpy and Entropy Contributions to Conformational Stability

**Figure 7:**
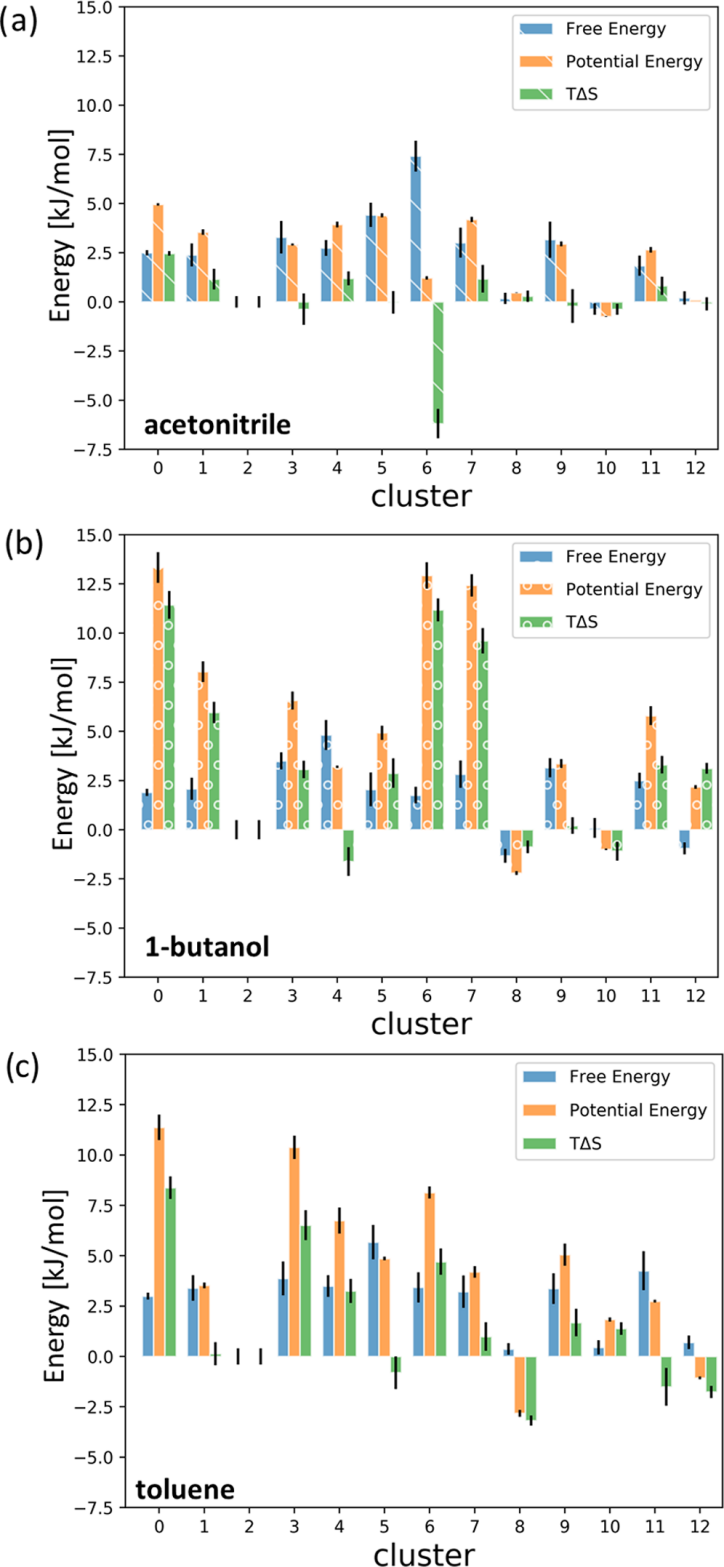
Relative free energy, potential energy, and entropy of each cluster configuration with respect to C2 for the case of (a) acetonitrile, (b) 1-butanol, and (c) toluene.

To gain further insight into the conformational isomerism of sildenafil in solution, the free energy Δ*G* of each state is calculated, along with a breakdown into potential energy and entropy. In Figure 7, the relative free energy of each state with respect to structure C2 for each solvent can be found in blue. As expected, the free energy difference between the four structures dominating the probability density plot—C2, C8, C10, and C12—is less than 1 kJ/mol for all solvents. The difference in free energy between the latter group and the rest of the structures varies between 2.5 and 5.5 kJ/mol, with the exception of C6 for the case of acetonitrile. Despite the minor variations in Δ*G* between different solvent cases, however, the potential energy and entropy reveal more significant differences induced by the solvent. The relative potential energy difference (with respect to C2) rarely exceeds 5 kJ/mol in the case of acetonitrile, which also translates into minor entropic contributions in most states. The exception is once again C6 for which the free energy is dominated by configurational entropy. A significantly different observation can be made for the cases of 1-butanol and toluene, where the relative potential energy of states is much larger, varying between 5 and 13 kJ/mol. Equally, entropy contributions in these two solvents are much more substantial than for the case of acetonitrile, indicating that solute–solvent interactions are much more dynamical. This is particularly prominent for the case of 1-butanol and it is possibly related to the inherent flexibility of its aliphatic chain.

## Conclusions

In this paper, we develop a systematic approach to partition the configuration space of flexible molecules with an arbitrary number of rotatable bonds into conformational macrostates. The approach is based on the development of a distance metric between configurations that incorporates qualitative information on the energetic cost associated with transitions along each degree of freedom, and the subsequent application of unsupervised clustering. We apply this approach to investigate the conformational landscape of sildenafil in the crystal bulk, in the gas phase, and in solution. A key aspect of the methodology introduced in this work is that the cluster centers are identified only once, for a reference state in the gas phase. These cluster centers configurations are then used to classify configurations sampled in solution. This approach provides a self-consistent identification scheme for clusters in the condensed phase. Using this methodology, 95% of structures in three different solvents are unambiguously assigned to a cluster, demonstrating the effectiveness of the proposed classification procedure. We demonstrate that this classification strategy can be coupled with reweighting strategies to compute the free energy of conformational states and to further decompose it into its enthalpy and entropy contributions. This analysis leads to new insights into the role of solvent in the definition of the conformational landscape of an organic molecule. It is found that, while the relative free energy variation between states in different solvents is limited, solvents cases 1-butanol and toluene cause an increase in the entropic contribution to the conformational free energy. Combining this approach with existing strategies for studying the effects of conformational rearrangements on processes can prove invaluable in understanding the effect of conformational isomerism in the process of crystal nucleation, growth, and dissolution for systems of any size and level of conformational complexity.

## Supporting Information Available

Sildenafil form I and form II crystal cell parameters; solvent density at ambient temperature (300 K) and pressure (1 bar); free energy profiles along torsional angles in the gas phase; conformational isomers in the crystal; and conformational fingerprints (PDF)
