# Supplementary material for: Identifying Conformational Isomers of Organic Molecules in Solution via Unsupervised Clustering
Source: J Chem Inf Model. 2021 Apr 29;61(5):2263–73. doi: 10.1021/acs.jcim.0c01387 (PMC8278389; doi:10.1021/acs.jcim.0c01387)
Supplement: Supplementary file 1 — ci0c01387_si_001.pdf [file ci0c01387_si_001.pdf]

Identifying conformational isomers of organic molecules in solution via unsupervised clustering.

Supplementary Information

Veselina Marinova,[†] Laurence Dodd,[†] Song-Jun Lee,[†] Geoffrey P. F. Wood,[¶] Ivan Marziano,[§] and Matteo Salvalaglio[*,†]

†*Thomas Young Centre and Department of Chemical Engineering, University College London, London WC1E 7JE, UK.*

‡*Department of Materials Science and Engineering, The University of Sheffield, Sheffield S1 3JD, UK*

¶*Pfizer Worldwide Research and Development, Groton Laboratories, Groton, Connecticut 06340, USA*

§*Pfizer Worldwide Research and Development, Sandwich, Kent CT13 9NJ, UK*

E-mail: m.salvalaglio@ucl.ac.uk

Force Field Validation

The following tables show a comparison between parameters obtained from the MD simulations performed in this study and experimental results reported in literature.

Table 1 and 2 report the crystal cell parameters obtained from our *unbiased* MD simulations in an isothermal-isobaric ensemble, where anisotropic pressure control was applied through the use of the Berendsen barostat. Both for forms I and II we observe good agreement with literature for the crystal cell parameters.

Table S1: Sildenafil Form I Crystal Cell Parameters

**Form I**	**Experiment**	**MD**
x [nm]	1.73	1.69
y [nm]	1.70	1.78
z [nm]	0.83	0.82
density [kg m^{-3}]	1298	1272

Table S2: Sildenafil Form II Crystal Cell Parameters

**Form II**	**Experiment**	**MD**
x [nm]	3.57	3.43
y [nm]	1.71	1.81
z [nm]	0.81	0.78
density [kg m^{-3}]	1273	1282

Solvent density was compared against the PubChem Database of the National Center for Biotechnology Information as shown in Table 3. For our production runs of *biased* MD, the Parrinello-Rahman barostat was used for 1-butanol and toluene, while the Berendsen barostat was employed in the case of acetonitrile, where Parrinello-Rahman displayed an unstable behavior. Table 3 reports on the solvent density obtained from the production runs in our *biased* MD simulations.

Table S3: Solvent Density at ambient temperature (300 K) and pressure (1 bar).

**Solvent**	**Experimental Density** [kg m^{-3}]	**MD density** [kg m^{-3}]
acetonitrile	783	734
1-butanol	810	823
toluene	862	871

To ensure that the Berendsen barostat is providing an adequate representation of the solvent density in the case of acetonitrile for our produciton runs we also compare the density when performing a 2 ns *unbiased* MD simulation against that of the Parrinello-Rahman barostat as showin in Table 4.

Table S4: Comparison between the density in acetonitrile of a 2 ns

**Solvent**	**Berendsen** [kg m^{-3}]	**Parrinello-Rahman** [kg m^{-3}]
acetonitrile	734 ±0.4	736 ±0.5

Free Energy Profiles along Torsional Angles in the Gas Phase

A free energy surface $F(\tau_i)$ for each ith torsional angle of sildenafil in the gas phase was obtained by Boltzmann inversion of the probability density estimated from MD. The standard error of the FES, associated with each free energy profile, was calculated using block averaging. The MD simulation trajectory (450 ns) was segmented in $n_B = 15$ blocks, each of $\sim$ 30 ns. The standard error of the free energy was calculated as $\epsilon_{F_i} = \frac{\sigma_{F_i}}{\sqrt{n_B}}$, where σ_{F_i} is the standard deviation of the free energy surface computed with the sampling generated in the n_B blocks.

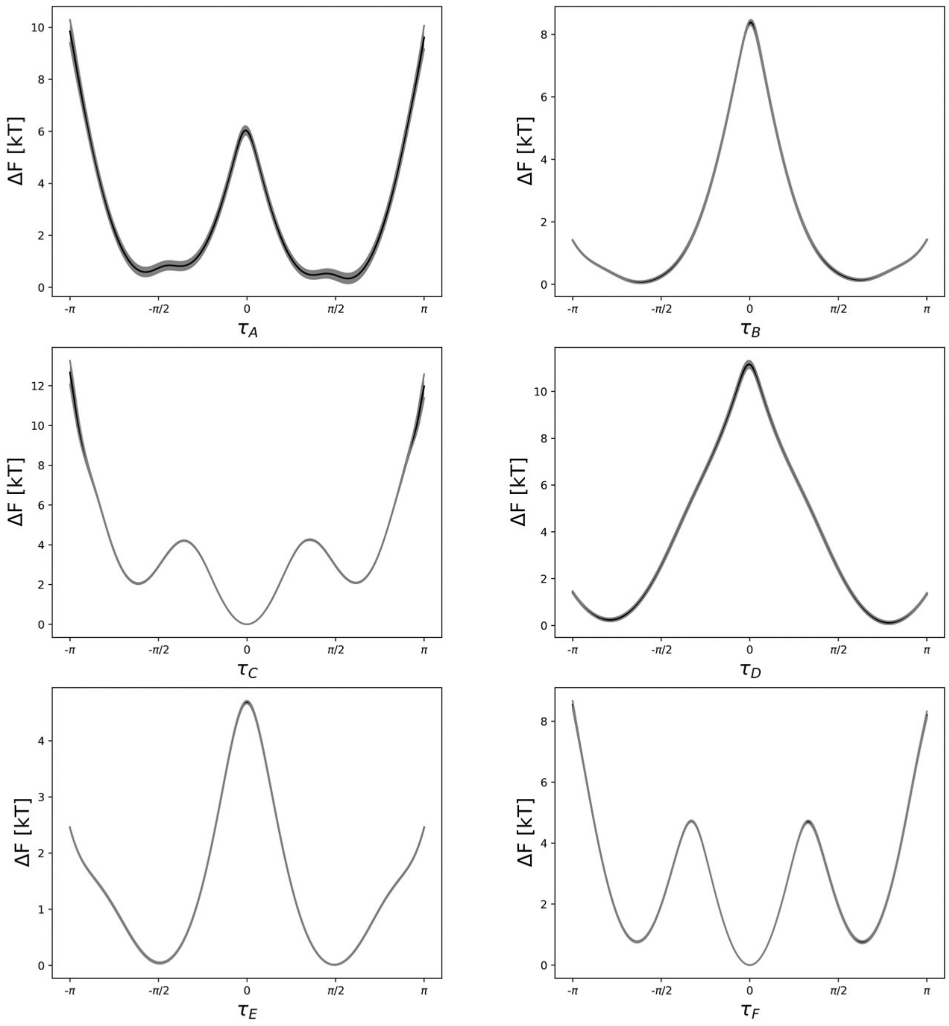

Figure S1: Free energy profiles of each torsional angle for a sildenafil molecule in the gas phase (black), along with the standard error in the free energy (dark gray).

Conformational Isomers in the Crystal

MD simulations of the two polymorphs (form I and form II) were performed to assess the conformational isomers of sildenafil present in each structure. In each crystal form two conformational isomers of sildenafil can be identified (conformer 1 and conformer 2). The conformers comparison between the two crystal forms has been reported in literature as conformational readjustment of the same gas-phase conformer. The probability distribution of each torsional angle of each conformer is shown in Figure S5. The plots show that within the same crystal forms, conformer 1 and conformer 2 are geometrical isomers, where rotation along τ_A and τ_E can convert one into the other. On the other hand, comparing conformers between different crystal structures, reveals only a slight difference in the distribution in torsional angle τ_E as discussed in the main text. To confirm the similarity in the conformers between the two polymorphs, a Hellinger distance of the given four distributions is computed, confirming that the difference in the conformational isomers between the two structures is minimal, which correlates with experimental findings.

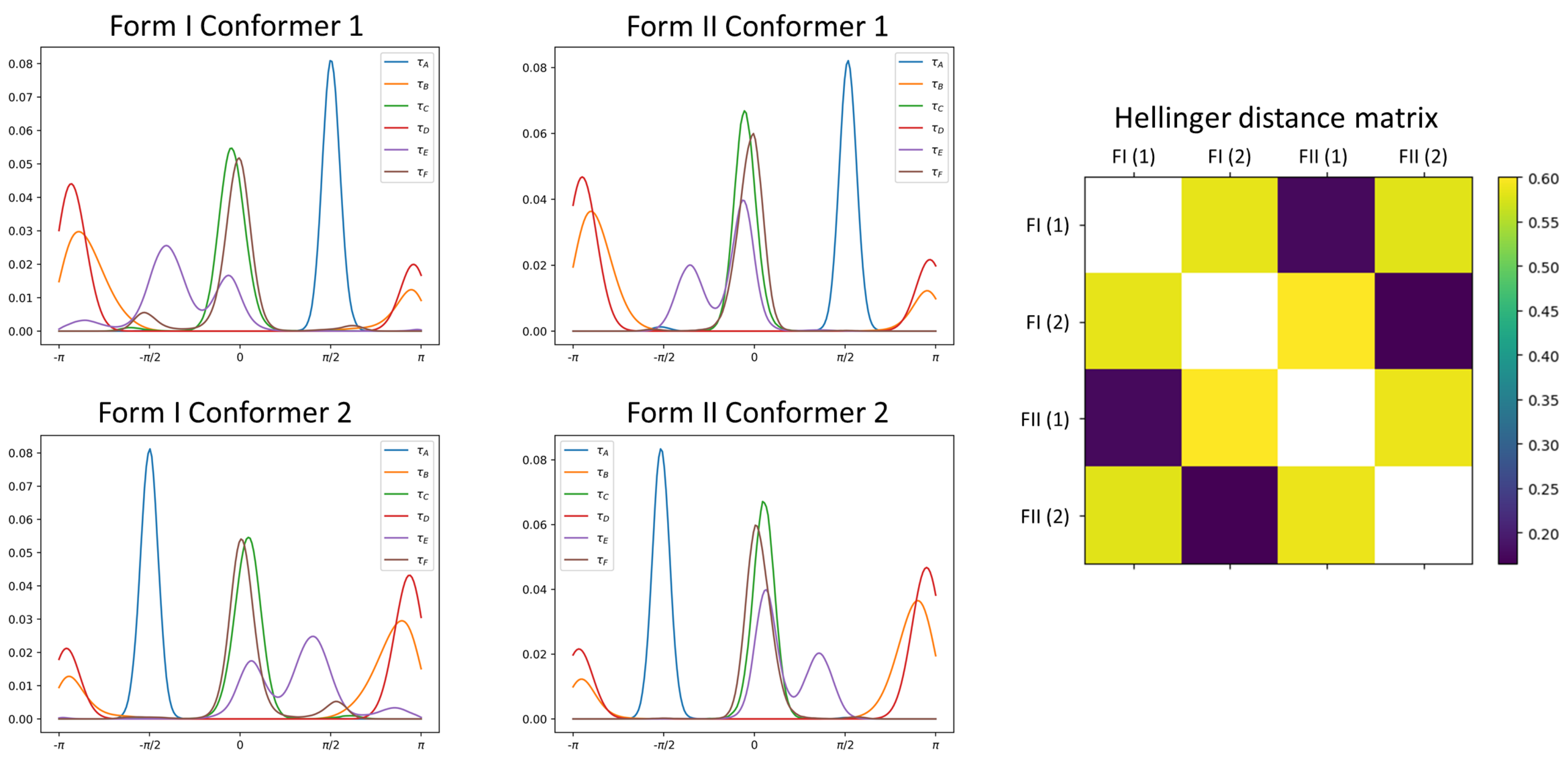

Figure S2: Comparison of sildenafil crystal conformers in the crystal bulk of form I and form II.

Conformational Fingerprints

The fingerprints used to identify conformational states of the molecule, which were generated using the FSFDP clustering algorithm, are shown here. Four of the distributions were post processed to ease the dimensionality reduction algorithm, discussed in Section D of the Methods, and therefore both pre- and post- processing figures are provided, indicated by an asterisk.

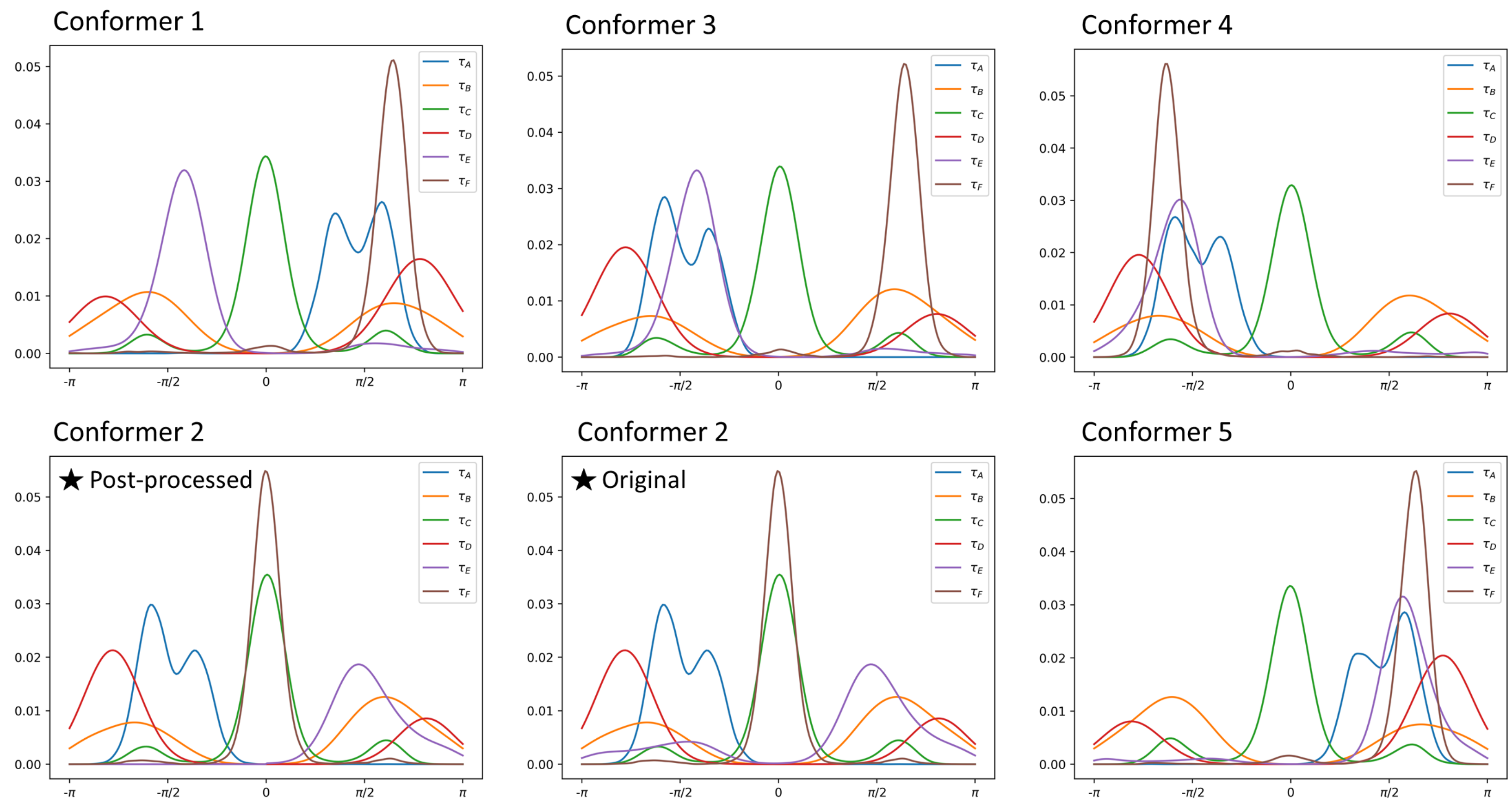

Figure S3: Torsional angle fingerprints of identified conformers

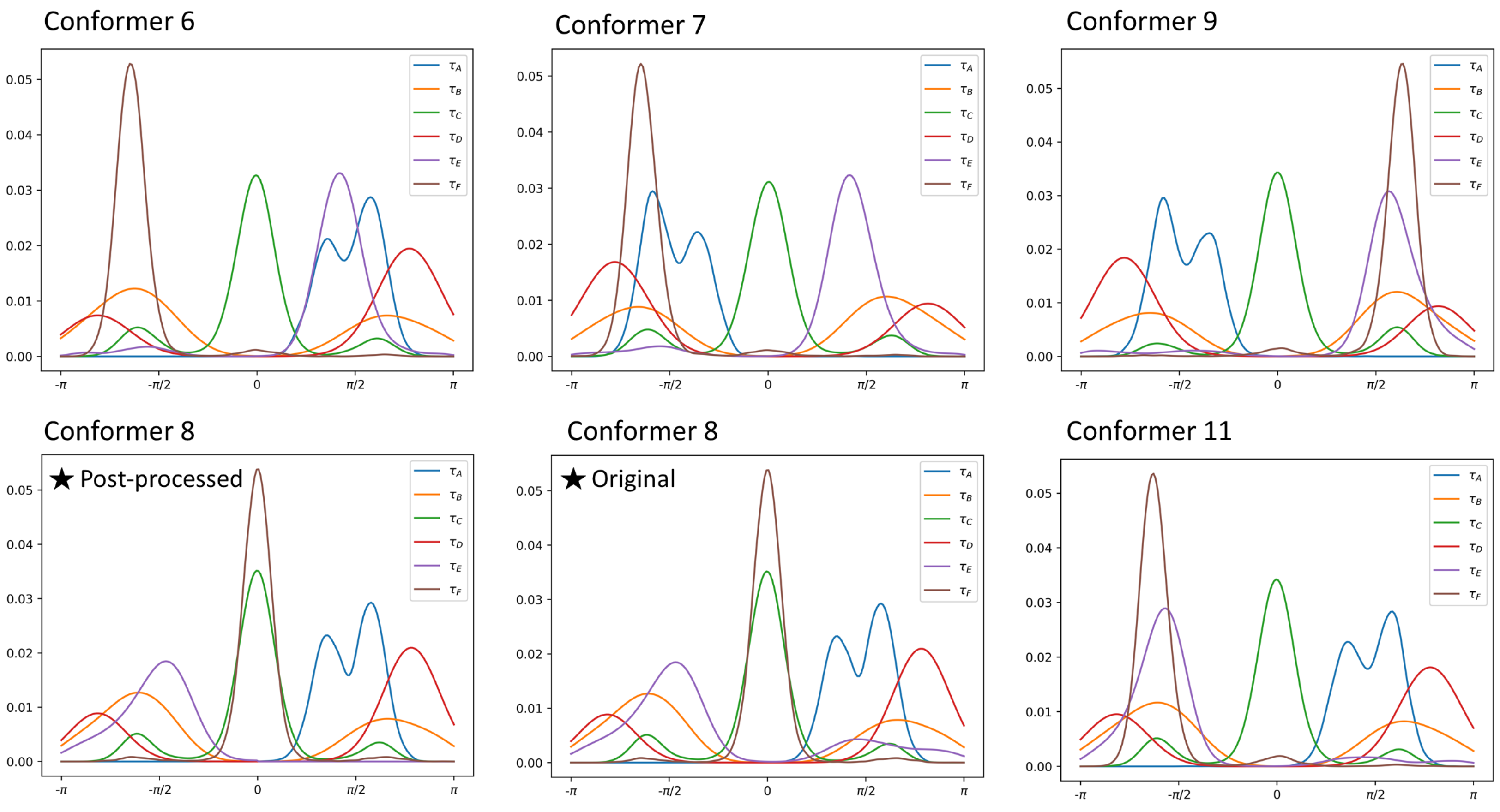

Figure S4: Torsional angle fingerprints of identified conformers

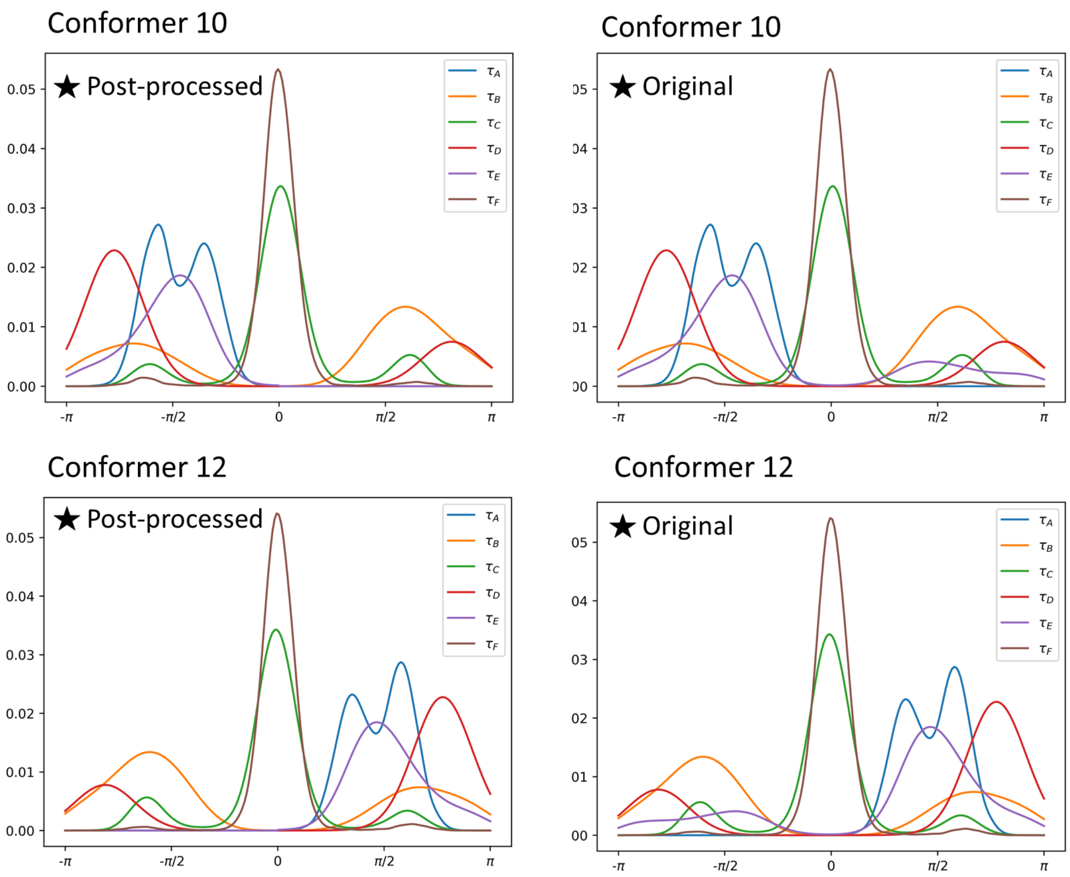

Figure S5: Torsional angle fingerprints of identified conformers
